# A Single Perioperative Injection of Dexamethasone Decreases Nausea, Vomiting, and Pain after Laparoscopic Donor Nephrectomy

**DOI:** 10.1155/2017/3518103

**Published:** 2017-01-22

**Authors:** Shigeyoshi Yamanaga, Andrew Mark Posselt, Chris Earl Freise, Takaaki Kobayashi, Mehdi Tavakol, Sang-Mo Kang

**Affiliations:** ^1^Division of Transplant Surgery, Department of Surgery, University of California San Francisco, San Francisco, CA, USA; ^2^Department of General Surgery, Japanese Red Cross Kumamoto Hospital, Kumamoto, Kumamoto, Japan; ^3^Department of Renal Transplant Surgery, Aichi Medical University School of Medicine, Nagakute, Aichi, Japan

## Abstract

*Background.* A single dose of perioperative dexamethasone (8–10 mg) reportedly decreases postoperative nausea, vomiting, and pain but has not been widely used in laparoscopic donor nephrectomy (LDN).* Methods.* We performed a retrospective cohort study of living donors who underwent LDN between 2013 and 2015. Donors who received a lower dose (4–6 mg)  (*n* = 70) or a higher dose (8–14 mg) of dexamethasone (*n* = 100) were compared with 111 donors who did not receive dexamethasone (control). Outcomes and incidence of postoperative nausea, vomiting, and pain within 24 h after LDN were compared before and after propensity-score matching.* Results*. The higher dose of dexamethasone reduced postoperative nausea and vomiting incidences by 28% (*P* = 0.010) compared to control, but the lower dose did not. Total opioid use was 29% lower in donors who received the higher dose than in control (*P* = 0.004). The higher dose was identified as an independent factor for preventing postoperative nausea and vomiting. Postoperative complication rates and hospital stays did not differ between the groups. After propensity-score matching, the results were the same as for the unmatched analysis.* Conclusion.* A single perioperative injection of 8–14 mg dexamethasone decreases antiemetic and narcotic requirements in the first 24 h, with no increase in surgical complications.

## 1. Introduction

Laparoscopic donor nephrectomy (LDN) has been proven to be a safe procedure [1–3] and is the gold standard for donor nephrectomy. LDN has many advantages compared to open donor nephrectomy, including reduced pain, earlier return to work, and improved cosmetic results [1]. However, donors continue to require significant amounts of antiemetics and opioids postoperatively.

Postoperative nausea and vomiting (PONV) occurs in 50–75% of patients after laparoscopic surgery [4]. One of the drugs for PONV, dexamethasone, is a long-acting corticosteroid known for its antiemetic and anti-inflammatory effects [5]. Numerous studies led to 4-5 mg dexamethasone being included as a first-line PONV prophylactic agent in several guidelines [6, 7] for perioperative anesthesia management. However, in other studies, a higher dose of dexamethasone (≥8 mg or ≥0.1 mg/kg) appeared more effective for PONV than the low dose of 4-5 mg [8, 9]. Furthermore, the higher dose is also effective for pain prophylaxis and the quality of recovery [4, 9–11], and these effects potentially accelerate the accomplishment of outpatient surgeries such as laparoscopic nephrectomy [12]. However, there were no studies regarding the efficacy of dexamethasone for PONV and pain after LDN.

Opioids play a central role in multimodal analgesia after LDN; however, side effects such as PONV, sedation, and reduced gastrointestinal motility [13] affect donor comfort and raise potentially serious safety concerns in this healthy population. These side effects have led to the use of the ketorolac in many centers, despite the low but finite risk of serious complications. Achieving donor comfort and safety while reducing opioids requires an alternative to ketorolac that would be effective and result in fewer adverse events.

In this retrospective analysis, we examined whether different doses of preoperative dexamethasone would be effective in reducing PONV and opioid consumption without increasing postoperative complications after LDN.

## 2. Materials and Methods

### 2.1. Patient Population

**Figure 1:**
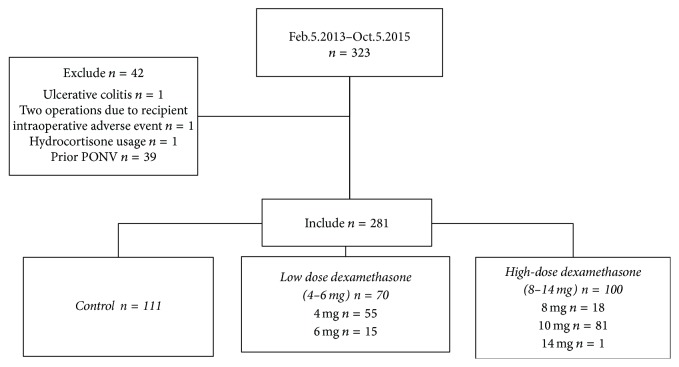
Flow chart of study design. PONV: postoperative nausea and vomiting.

We retrospectively evaluated the records of 323 consecutive donors who underwent LDN from February 5, 2013, to October 5, 2015, at the University of California, San Francisco. Of these, 42 donors were excluded from the analysis for the following reasons: one had a history of ulcerative colitis, one had two operations because the recipient had intraoperative problems during the first operation (when the first LDN was taking place), one received hydrocortisone instead of dexamethasone, and 39 had a prior history of PONV (confirmed by anesthesiologists by preoperative assessment; nine control, 12 low dose, and 18 high dose, *P* = 0.14), which is a well-established risk factor for PONV [14] and therefore should be excluded from outcome analysis [15]. Thus, we compared 70 donors who received a single intraoperative low dose (4–6 mg) and 100 donors who received a single intraoperative high dose (8–14 mg) of dexamethasone to 111 controls who received no dexamethasone (Figure 1).

### 2.2. Data Collection and Perioperative Management of Donors

Demographic data were collected from patients' electronic medical records. All donors ceased using tobacco at least six weeks before LDN. Bowel preparation was done using bisacodyl oral tablet 5 mg daily for three days prior to surgery. Dexamethasone was administered at the beginning of LDN according to a request by the anesthesiologist and/or surgeon. All donors received standard general anesthesia without epidural analgesia. All nephrectomies were done using the intraperitoneal pure LDN approach as previously published [3]. Incisions were as follows: four 5–12 mm working ports in the right or left mid abdomen and a Pfannenstiel incision (5–8 cm) made at the end of the LDN for the kidney extraction. Local anesthesia (bupivacaine, 0.25%, up to 1 cc/kg) was injected into all incisions at the end of the LDN, and patient-controlled analgesia was used for the first 24 h after LDN. Postoperative time was defined as the 24 h period after the anesthesiologist was no longer in personal attendance.

PONV is difficult to quantitate, particularly in a retrospective approach. We therefore used administration of antiemetics as an objective, surrogate marker for PONV. Notably, previous prospective studies utilizing subjective PONV scales have demonstrated that administration of antiemetics is highly correlated with nausea and vomiting [4, 10]. Ondansetron was the first choice of antiemetic drug for PONV episodes. We recorded the number of antiemetic injections during the first 24 h after LDN. Intravenous opiate analgesics included fentanyl and hydromorphone and were given during the first 24 h. Donors generally had conversion to oral analgesics between 6 h and 24 h after LDN. Total opiate consumption was converted into an oral morphine equianalgesic dose [16] and pooled for each of the three groups. Some patients received ketorolac and/or acetaminophen. Thus, the doses of all three analgesics (opioid, ketorolac, and acetaminophen) during the first 24 h after LDN were recorded. The usage of ketorolac and acetaminophen and their effects on narcotic consumption were also analyzed.

This study was approved by the Institutional Review Board of the University of California, San Francisco (study approval number 15-18306). This was a retrospective study of the drugs used for known clinical indications; therefore consent was not obtained.

### 2.3. Statistical Analysis

Values are expressed as mean ± standard deviation unless otherwise specified. The chi-squared test and Fisher's exact test were used to analyze categorical data, and Student's *t*-test, the Mann-Whitney *U* test, and the Kruskal-Wallis test were used to analyze continuous data. Bonferroni correction was used for post hoc analysis. Two-tailed *P* values < 0.05 were considered statistically significant.

Univariate and multivariate logistic regression analyses using pre- and postoperative variables were used to investigate whether dexamethasone administration was an independent variable in PONV. Specific variables associated with *P* values < 0.10 by univariate analysis were entered into multivariate analysis. The adequacy of the multivariate model was evaluated using the Hosmer and Lemeshow test.

To further validate the results of this retrospective study, propensity-score matching [17] was done on preoperative variables (age, sex, and body mass index (BMI)) between the control group and the high-dose dexamethasone group. Matching was done using the 1 : 1 method between ±0.01 differences.

All data were analyzed using SPSS version 23 (SPSS, Chicago, Illinois, USA). The authors have followed the suggestions of the Strengthening the Reporting of Observational Studies in Epidemiology (STROBE) statement guidelines for reporting observational studies [18].

## 3. Results

### 3.1. Donor Baseline Variables and Operative Outcomes

Donor baseline variables and operative outcomes are shown in Table 1. As expected, the groups were very similar, except the low dose dexamethasone group that had more women than the control or high-dose dexamethasone group. This was in general due to a propensity of some anesthesiologists to give low dose dexamethasone intraoperatively to women who are at increased risk of PONV [14]. Postoperative hospital stays and complications did not differ among the groups. There were no complications in the high-dose group, three in the low dose group (4.3%; 2 urinary retention, 1 headache), and three in the control group (2.7%; 1 high-grade fever with diarrhea, 1 surgical site hematoma, and 1 superficial surgical site infection requiring bed-side drainage and antibiotics). No complications were severe; all were below grade II of the Clavien-Dindo classification [19].

### 3.2. Dexamethasone Use and PONV

**Figure 2:**
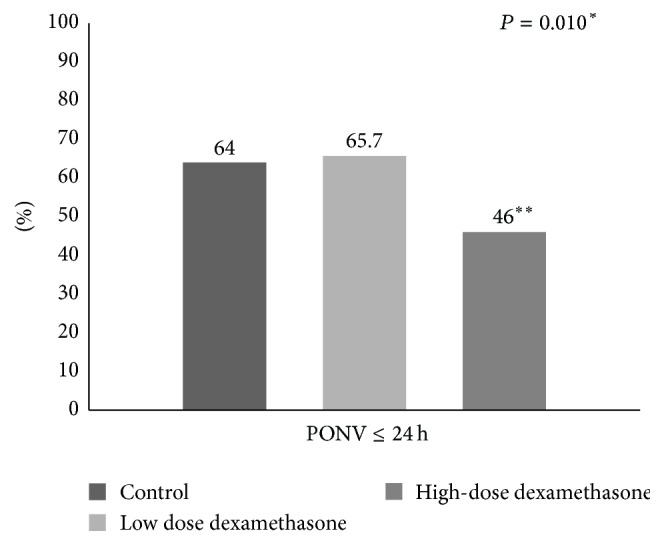
The incidence of postoperative nausea and vomiting. ^*∗*^Chi-squared test. ^*∗∗*^Statistically significant by post hoc analysis. PONV: postoperative nausea and vomiting.

High-dose dexamethasone significantly reduced the incidence of PONV by 28% more than control but the low dose did not (Figure 2). Over 50% of PONV episodes, measured by the number of times antiemetics were injected, appeared to occur within 6 h after LDN. Only high-dose dexamethasone reduced the number of PONV episodes (*P* = 0.039, post hoc analysis control versus high dose *P* = 0.044).

### 3.3. Univariate and Multivariate Analyses of Factors Associated with PONV

Female sex, low BMI, longer operative time, a single injection of high-dose dexamethasone, and the amount of total opioid were each associated with PONV on univariate analysis (Table 2). Female sex, low BMI, and a single injection of high-dose dexamethasone were independent factors associated with PONV on multivariate analysis, while low dose dexamethasone was not (Table 2).

### 3.4. Postoperative Pain and Intravenous Opioid Doses within 24 h

**Figure 3:**
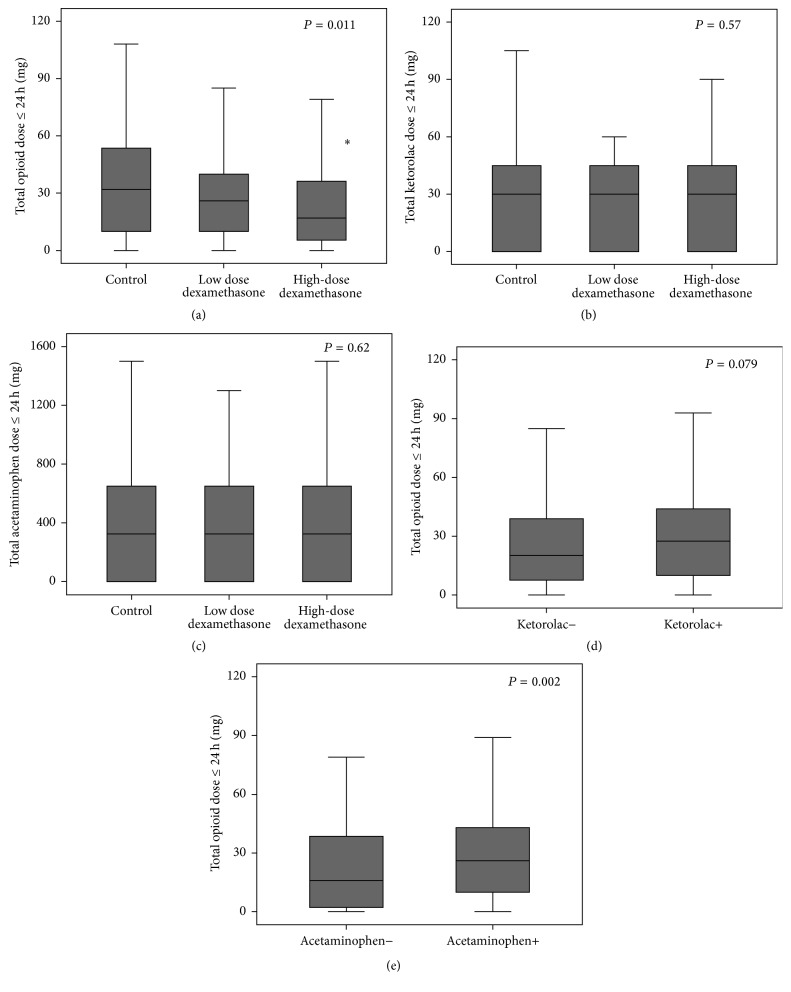
Box-and-whisker plot graphs showing total postoperative dose (≤24 h) of opioids (a), ketorolac (b), and acetaminophen (c) and subgroup analyses of opioid consumption with or without ketorolac ≤24 h (d) and with or without acetaminophen ≤24 h (e). Error bars indicate the highest and lowest occurring value within a 1.5 interquartile range. ^*∗*^Post hoc analysis control versus high dose *P* = 0.008 (adjusted).

Total postoperative opioid, ketorolac, and acetaminophen consumption with ≤24 h are shown in Figure 3. Over 90% of intravenous opioids were injected ≤6 h after LDN. A single injection of high-dose dexamethasone significantly reduced total opioid consumption ≤24 h [35.2 ± 29.8 mg (control) versus 27.8 ± 19.6 (low dose) versus 25.1 ± 28.0 mg (high dose); *P* = 0.011, post hoc analysis control versus high dose *P* = 0.008, Figure 3(a)].

The total dose of ketorolac and acetaminophen did not differ between the groups (Figures 3(b) and 3(c)). Among patients who received ketorolac and/or acetaminophen, postoperative opioid use increased slightly [26.9 ± 27.5 mg (without ketorolac) versus 31.9 ± 27.6 mg (with ketorolac), *P* = 0.079, Figure 3(d); 24.7 ± 26.7 mg (without acetaminophen) versus 31.9 ± 27.8 mg (with acetaminophen), *P* = 0.002, Figure 3(e)].

### 3.5. Propensity-Score Matching Analysis between Control and High-Dose Dexamethasone

After propensity-score matching for age, sex, and BMI between the control and the higher-dexamethasone groups, 81 matched pairs were obtained (Table 3). The results were the same as for the unmatched analysis in terms of PONV incidence ≤24 h [69.1% (control) versus 43.2% (high dose), *P* = 0.001] and total opioid consumption ≤24 h [39.6 ± 31.4 mg (control) versus 25.8 ± 29.2 mg (high dose), *P* = 0.008]. The total dose of ketorolac and acetaminophen did not differ between the two groups.

## 4. Discussion

Laparoscopic donor nephrectomy is now a widely accepted procedure and appears to be as safe as open donor nephrectomy. However, improving donor comfort postoperatively and preventing narcotic-related complications remains a primary, important goal. Our study is the first to show that a single higher (8–14 mg) dose of dexamethasone decreases the use of antiemetics and narcotics after LDN in a statistically and clinically significant manner. Specifically, the higher dose of dexamethasone reduced PONV incidence by 28% and total opioid use by 29% compared to control, and those efficacies were further confirmed by multivariate analysis and propensity-score matching.

However, general concerns about wound healing or infection in donors, a healthy patient population, may have prevented dexamethasone from being widely adopted, although ample evidence indicates that even higher doses of steroids do not increase wound complications in other types of surgeries [20]. Our analyses also clearly showed that higher dose dexamethasone reduces antiemetic and narcotic consumption without increasing the incidence of short-term complications, results that corroborate those of other studies [4, 9–11].

The magnitude of opioid-sparing effect of high-dose dexamethasone in our study (29%) is in line with previously published studies [9, 11] (15–50%), and it is in general higher than for ketorolac [21, 22] (6–22%) or acetaminophen [23] (16%). Furthermore, the evidence that ketorolac reduces opioid usage after LDN is still lacking. Ketorolac is a nonsteroidal anti-inflammatory drug [24] that is widely used in many transplant centers during LDN [25]. A retrospective study of open donor nephrectomies reported a 58% opioid reduction in patients who received ketorolac [26]. Although it was one of the landmark studies regarding the opioid-sparing effect of ketorolac in the field of donor nephrectomy, that study had serious confounders such as the use of a historical control despite the introduction of a new fast-track pathway for rapid discharge (intravenous hydration, encouraging early ambulation, and diet progression) in addition to ketorolac. In fact, several studies [25], including one randomized trial in patients undergoing LDN [27], showed no benefit of ketorolac on opioid consumption. Consistent with the results of those studies, our patients who received ketorolac did not have reductions in antiemetic or narcotic requirements. Thus, the routine use of ketorolac appears to be unsupported for LDN [24, 28, 29]. As with ketorolac, we did not find a significant impact of acetaminophen on reducing opioid consumption. Acetaminophen has no nephrotoxicity and is less harmful than ketorolac but may lead to having liver failure even with a therapeutic dose [30]. Further studies are needed to confirm the opioid-sparing effect of these medications in the LDN population.

We recognize that a major limitation of our study is that it is a retrospective analysis of a single center. However, several factors support the conclusions of our study. First, the donor nephrectomy patients are otherwise healthy and represent a relatively homogeneous group of patients (in terms of baseline characteristics including unknown variables) compared to those undergoing other types of major surgery, and also LDN was performed in a standardized manner. Notably, the magnitude of decrease in PONV was similar when controlling for surgeon, although the smaller numbers preclude robust statistical differences (data not shown). The lower dose dexamethasone had more women who are at increased risk of PONV [14]. However, we attempted to mitigate this bias using multivariate logistic regression analysis showing that, even after the adjustment, the lower dose still had no impact on preventing PONV in this population. Furthermore, the day to day postoperative care was similar for all patients regardless of surgeon or dexamethasone administration, as the inpatient team follows a standard postoperative routine for donors. Second, when we performed propensity-score matching to help mitigate the biases introduced by retrospective studies, our results were essentially the same. Third, the magnitude of decrease in PONV and narcotic usage was substantial and well supported by multiple trials in other types of surgeries, as well as meta-analyses [4, 9–11]. Although we excluded donors with a prior history of PONV from the analysis, the efficacy of high-dose dexamethasone compared to control followed the same pattern if we included those for analyses (data not shown). Thus, while our study design had limitations, we believe our conclusions have a high likelihood of validity.

In conclusion, our study shows that a single perioperative injection of 8–14 mg dexamethasone decreased antiemetic and narcotic requirements in the first 24 h after LDN, with no increase in short-term complications. This translates into improved donor comfort and potentially improved safety through fewer complications associated with narcotic use. Moreover, dexamethasone is widely available and inexpensive. Thus we believe that it is justified for use in virtually all laparoscopic donors, as is now the standard at our institution.

## Figures and Tables

**Table 1:** Donor baseline variables and surgical outcomes.

	Control (*n* = 111)	Low dose dexamethasone (*n* = 70)	High-dose dexamethasone (*n* = 100)	*P* values	*P* values
				Control versus low dose	Control versus high dose
Age (years)	43.6 ± 13.4	41.7 ± 13.0	40.5 ± 12.5	0.34	0.09
Sex				<0.001	0.56
Male	51 (45.9)	13 (18.6)	42 (42.0)		
Female	60 (54.1)	57 (81.4)	58 (58.0)		
BMI (kg/m^2^)	26.5 ± 3.4	25.8 ± 4.1	26.7 ± 3.7	0.31	0.65
ASA				0.18	0.21
1	77 (69.4)	55 (78.6)	77 (77.0)		
2	34 (30.6)	15 (21.4)	23 (23.0)		
Intraoperative ondansetron use	103 (92.8)	64 (91.4)	93 (93.0)	0.74	0.95
Operative time (min)	147.8 ± 25.0	148.1 ± 22.7	155.4 ± 24.1	0.93	0.009
Side of nephrectomy				0.013	0.85
Left	91 (81.1)	46 (65.7)	82 (82.0)		
Right	20 (18.9)	24 (34.3)	18 (18.0)		
Postoperative hospital stay (days)	2.68 ± 0.62	2.63 ± 0.59	2.66 ± 0.62	0.69	0.86
Complications (%)	3 (2.7)	3 (4.3)	0 (0)	0.68	0.25

Data are expressed as mean ± SD or *n* (%).

ASA: American Society of Anesthesiologists; BMI: body mass index.

**Table 2:** Univariate and multivariate analyses of factors associated with postoperative nausea and vomiting.

	Univariate logistic regression analysis			Multivariate logistic regression analysis^†^		
	OR	95% CI	*P* value	OR	95% CI	*P* value
Age (per decade)	0.95	0.80–1.14	0.61			
Sex (ref. male)	2.81	1.71–4.62	<0.001	2.78	1.60–4.83	<0.001
BMI (per 1 kg/m^2^)	0.91	0.85–0.98	0.007	0.91	0.85–0.98	0.012
Side of kidney (ref. left)	0.96	0.54–1.68	0.88			
ASA (ref. 1)	0.81	0.47–1.39	0.44			
Operative time (per 60 min)	0.65	0.41–1.03	0.068	1.03	0.61–1.73	0.91
Dexamethasone (ref. control)			0.011			0.041
Low dose	1.08	0.58–2.02	0.81	0.81	0.41–1.60	0.55
High dose	0.48	0.28–0.83	0.009	0.47	0.26–0.86	0.014
Intraoperative ondansetron use	0.53	0.20–1.41	0.20			
Ketorolac use (ref. no use)	1.01	0.63–1.63	0.96			
Acetaminophen use (ref. no use)	1.43	0.85–2.40	0.17			
Total opioid (per 1 mg)	1.01	1.00–1.02	0.035	1.01	1.00–1.02	0.058

^†^Hosmer and Lemeshow Test: *P* value 0.30

ASA: American Society of Anesthesiologists; BMI: body mass index; CI: confidential interval; OR: odds ratio.

**Table 3:** Donor baseline variables and surgical outcomes in propensity-score matched cohort.

	Control (*n* = 81)	High-dose dexamethasone (*n* = 81)	*P* value
Age (years)	41.6 ± 13.2	41.1 ± 11.9	0.84
Sex			0.87
Male	32 (39.5)	33 (40.7)	
Female	49 (60.5)	48 (59.3)	
BMI (kg/m^2^)	26.6 ± 3.3	26.8 ± 3.8	0.70
ASA			0.49
1	56 (69.1)	60 (74.1)	
2	25 (30.9)	21 (25.9)	
Intraoperative ondansetron use	75 (92.6)	74 (91.4)	0.77
Operative time (min)	140.1 ± 19.5	154.3 ± 22.6	<0.001
Side of nephrectomy			1.00
Left	65 (80.2)	65 (80.2)	
Right	16 (19.8)	16 (19.8)	
Postoperative hospital stay (days)	2.73 ± 0.63	2.62 ± 0.62	0.29
Complications (%)	3 (3.7)	0 (0)	0.25

Data are expressed as mean ± SD or *n* (%).

ASA: American Society of Anesthesiologists; BMI: body mass index.

## Abbreviations

ASA: American Society of Anesthesiologists
BMI: Body mass index
CI: Confidential interval
LDN: Laparoscopic donor nephrectomy
OR: Odds ratio
PONV: Postoperative nausea and vomiting.

## References

[1] Matas A. J., Bartlett S. T., Leichtman A. B., Delmonico F. L. (2003). Morbidity and mortality after living kidney donation, 1999-2001: survey of United States transplant centers. *American Journal of Transplantation*.

[2] Troppmann C., Perez R. V., McBride M. (2008). Similar long-term outcomes for laparoscopic versus open live-donor nephrectomy kidney grafts: an OPTN database analysis of 5532 adult recipients. *Transplantation*.

[3] Ahearn A. J., Posselt A. M., Kang S.-M., Roberts J. P., Freise C. E. (2011). Experience with laparoscopic donor nephrectomy among more than 1000 cases: low complication rates, despite more challenging cases. *Archives of Surgery*.

[4] Karanicolas P. J., Smith S. E., Kanbur B., Davies E., Guyatt G. H. (2008). The impact of prophylactic dexamethasone on nausea and vomiting after laparoscopic cholecystectomy: a systematic review and meta-analysis. *Annals of Surgery*.

[5] Callery M. P. (2003). Preoperative steroids for laparoscopic surgery. *Annals of Surgery*.

[6] Apfelbaum J. L., Silverstein J. H., Chung F. F. (2013). Practice guidelines for postanesthetic care: an updated report by the American Society of Anesthesiologists Task Force on postanesthetic care. *Anesthesiology*.

[7] Gan T. J., Diemunsch P., Habib A. S. (2014). Consensus guidelines for the management of postoperative nausea and vomiting. *Anesthesia and Analgesia*.

[8] Elhakim M., Nafie M., Mahmoud K., Atef A. (2002). Dexamethasone 8 mg in combination with ondansetron 4 mg appears to be the optimal dose for the preventiion of nausea and vomiting after laparoscopic cholecystectomy. *Canadian Journal of Anesthesia*.

[9] De Oliveira G. S., Ahmad S., Fitzgerald P. C. (2011). Dose ranging study on the effect of preoperative dexamethasone on postoperative quality of recovery and opioid consumption after ambulatory gynaecological surgery. *British Journal of Anaesthesia*.

[10] Sánchez-Rodríguez P.-E., Fuentes-Orozco C., González-Ojeda A. (2010). Effect of dexamethasone on postoperative symptoms in patients undergoing elective laparoscopic cholecystectomy: randomized clinical trial. *World Journal of Surgery*.

[11] Thangaswamy C. R., Rewari V., Trikha A., Dehran M., Chandralekha (2010). Dexamethasone before total laparoscopic hysterectomy: a randomized controlled dose-response study. *Journal of Anesthesia*.

[12] Azawi N. H., Christensen T., Dahl C., Lund L. (2016). Laparoscopic nephrectomy as outpatient surgery. *Journal of Urology*.

[13] Wu C. L., Raja S. N. (2011). Treatment of acute postoperative pain. *The Lancet*.

[14] Apfel C. C., Heidrich F. M., Jukar-Rao S. (2012). Evidence-based analysis of risk factors for postoperative nausea and vomiting. *British Journal of Anaesthesia*.

[15] Rawlins K., Kessell G. (2010). Postoperative nausea and vomiting in paediatric strabismus surgery. *British Journal of Anaesthesia*.

[16] Mcpherson M. L. (2010). *Demystifying Opioid Conversion Calculations: A Guide for Effective Dosing*.

[17] Baek S., Park S. H., Won E., Park Y. R., Kim H. J. (2015). Propensity score matching: a conceptual review for radiology researchers. *Korean Journal of Radiology*.

[18] von Elm E., Altman D. G., Egger M., Pocock S. J., Gøtzsche P. C., Vandenbroucke J. P. (2007). The Strengthening the Reporting of Observational Studies in Epidemiology (STROBE) statement: guidelines for reporting observational studies. *Lancet*.

[19] Dindo D., Demartines N., Clavien P.-A. (2004). Classification of surgical complications: a new proposal with evaluation in a cohort of 6336 patients and results of a survey. *Annals of Surgery*.

[20] Eberhart L. H. J., Holdorf S., Albert U. S. (2011). Impact of a single perioperative dose of dexamethasone on the incidence of surgical site infections: a case-control study. *The Journal of Obstetrics and Gynaecology Research*.

[21] Manworren R. C. B., McElligott C. D., Deraska P. V. (2016). Efficacy of analgesic treatments to manage children's postoperative pain after laparoscopic appendectomy: retrospective medical record review. *AORN Journal*.

[22] De Oliveira G. S., Agarwal D., Benzon H. T. (2012). Perioperative single dose ketorolac to prevent postoperative pain: a meta-analysis of randomized trials. *Anesthesia and Analgesia*.

[23] Mcnicol E. D., Ferguson M. C., Haroutounian S., Carr D. B., Schumann R. (2016). Single dose intravenous paracetamol or intravenous propacetamol for postoperative pain. *Cochrane Database of Systematic Reviews*.

[24] Ready L. B., Brown C. R., Stahlgren L. H. (1994). Evaluation of intravenous ketorolac administered by bolus or infusion for treatment of postoperative pain: a double-blind, placebo-controlled, multicenter study. *Anesthesiology*.

[25] Breda A., Bui M. H., Liao J. C., Schulam P. G. (2007). Association of bowel rest and ketorolac analgesia with short hospital stay after laparoscopic donor nephrectomy. *Urology*.

[26] Freedland S. J., Blanco-Yarosh M., Sun J. C. (2002). Ketorolac-based analgesia improves outcomes for living kidney donors. *Transplantation*.

[27] Grimsby G. M., Conley S. P., Trentman T. L. (2012). A double-blind randomized controlled trial of continuous intravenous ketorolac vs placebo for adjuvant pain control after renal surgery. *Mayo Clinic Proceedings*.

[28] Feldman H. I., Kinman J. L., Berlin J. A. (1997). Parenteral ketorolac: the risk for acute renal failure. *Annals of Internal Medicine*.

[29] García Rodríguez L. A., Cattaruzzi C., Troncon M. G., Agostinis L. (1998). Risk of hospitalization for upper gastrointestinal tract bleeding associated with ketorolac, other nonsteroidal anti-inflammatory drugs, calcium antagonists, and other antihypertensive drugs. *Archives of Internal Medicine*.

[30] Pogatzki-Zahn E., Chandrasena C., Schug S. A. (2014). Nonopioid analgesics for postoperative pain management. *Current Opinion in Anaesthesiology*.

